# Critical Roles of Kupffer Cells in the Pathogenesis of Alcoholic Liver Disease: From Basic Science to Clinical Trials

**DOI:** 10.3389/fimmu.2016.00538

**Published:** 2016-11-29

**Authors:** Tao Zeng, Cui-Li Zhang, Mo Xiao, Rui Yang, Ke-Qin Xie

**Affiliations:** ^1^Institute of Toxicology, School of Public Health, Shandong University, Jinan, China

**Keywords:** Kupffer cells, alcoholic liver disease, lipopolysaccharide, polarization, tumor necrosis factor α, cytochrome P4502E1

## Abstract

Alcoholic liver disease (ALD) encompasses a spectrum of liver injury ranging from steatosis to steatohepatitis, fibrosis, and finally cirrhosis. Accumulating evidences have demonstrated that Kupffer cells (KCs) play critical roles in the pathogenesis of both chronic and acute ALD. It has become clear that alcohol exposure can result in increased hepatic translocation of gut-sourced endotoxin/lipopolysaccharide, which is a strong M1 polarization inducer of KCs. The activated KCs then produce a large amount of reactive oxygen species (ROS), pro-inflammatory cytokines, and chemokines, which finally lead to liver injury. The critical roles of KCs and related inflammatory cascade in the pathogenesis of ALD make it a promising target in pharmaceutical drug developments for ALD treatment. Several drugs (such as rifaximin, pentoxifylline, and infliximab) have been evaluated or are under evaluation for ALD treatment in randomized clinical trials. Furthermore, screening pharmacological regulators for KCs toward M2 polarization may provide additional therapeutic agents. The combination of these potentially therapeutic drugs with hepatoprotective agents (such as zinc, melatonin, and silymarin) may bring encouraging results.

## Introduction

Alcoholic liver disease (ALD) remains one of the predominant causes of liver-related morbidity and mortality worldwide (1). ALD encompasses a spectrum of progressively aggregated liver diseases, from simple steatosis, to steatohepatitis, fibrosis, and finally cirrhosis (2, 3). It has been generally accepted that ALD is a multifactorial disease, and both parenchymal cells (hepatocytes) and non-parenchymal cells in the liver are involved in the pathogenesis of ALD. Accumulating evidence suggests that Kupffer cells (KCs), the resident macrophages in the liver, play crucial roles (4–6). KCs originate from bone narrow-derived monocytes and account for about 20–25% of non-parenchymal cells in the liver (7). KCs play key roles in host defense by removing foreign, toxic and infective substances from the portal blood and have been demonstrated to be involved in the pathogenesis of many kinds of liver diseases (8, 9). It has been demonstrated that KCs are activated by gut-derived endotoxin/lipopolysaccharide (LPS), and then release many hepatotoxicants including reactive oxygen species (ROS), tumor necrosis factor α (TNF-α), interleukins, and chemoattractants for cytotoxic neutrophils, which will impair the function and viability of the neighboring cells. The “gut–liver axis” theory provides a number of potential therapeutic targets for ALD treatment, which have been evaluated or are under evaluation in clinical trials (10, 11). Furthermore, KCs are exceptionally plastic cells that can polarize to specific activation states and express different functions in different microenvironment. Two extremes of macrophage polarization have been designated as M1 (classically activation) and M2 (alternative activation). M1-polarized KCs can produce a large amount of pro-inflammatory cytokines, such as TNF-α, while M2-polarized KCs exhibit high expression of anti-inflammatory mediators, such as interleukin 10 (IL-10) (4, 12). Results of recent studies suggest that pharmacological intervention targeting M2 KCs polarization may represent an attractive strategy for ALD treatment (5). In this review, we discuss the critical roles of KCs in the pathogenesis of both chronic and acute ALD, KCs polarization in ALD, and potential therapeutic targets for ALD treatment.

## Critical Roles of KCs in the Pathogenesis of Chronic ALD

**Figure 1:**
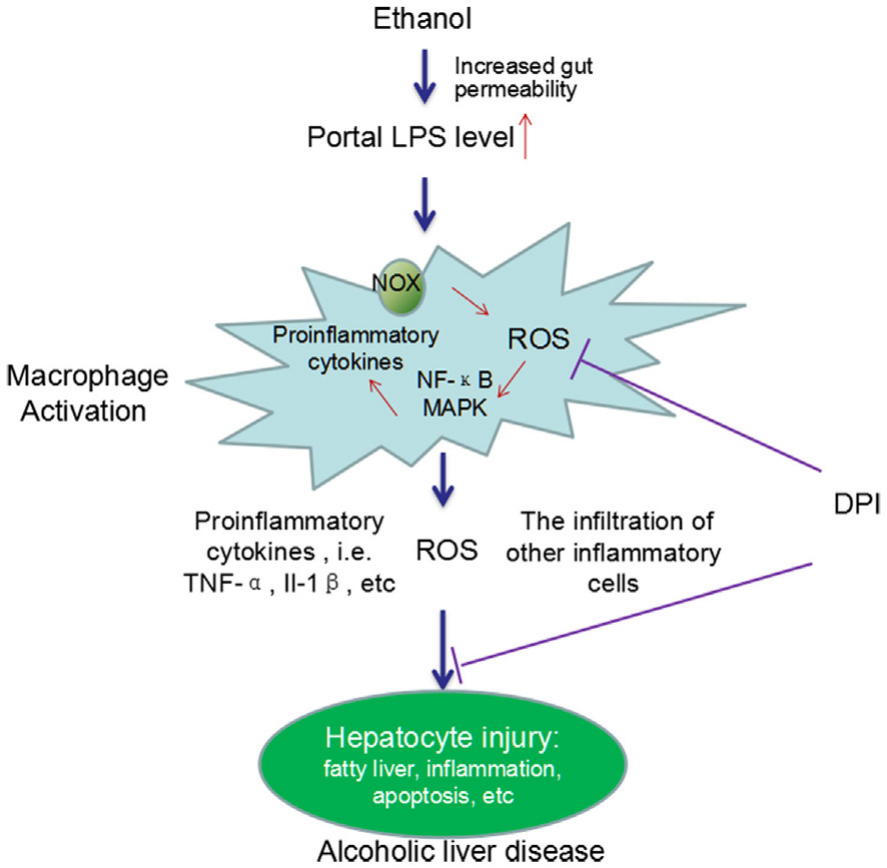
**A schema graph for the critical roles of KCs in the pathogenesis of ALD**. Chronic ethanol exposure increases the gut permeability, resulting in translocation of gut endotoxin/LPS to liver. In liver, LPS leads to KCs activation *via* activating NOX and the TLR-4 pathway. Activated KCs produce a large amount of ROS, pro-inflammatory cytokines, and chemokines and induce the infiltration of other inflammatory cells. The ROS, pro-inflammatory cytokines, and the infiltration of other inflammatory cells finally cause liver injury. DPI, a NOX inhibitor, can significantly block ethanol-induced oxidative stress and the liver injury. In addition, chronic ethanol exposure can also sensitize the LPS-induced toxicity by increasing the half-life and cell surface receptor number of TNF-α, the expression of TLR-related co-receptors, and forming oxidative stress-related pro-inflammatory adducts, such as MAA adducts (MDA reacts with acetaldehyde and proteins to form hybrid protein adducts).

Considerable evidence has demonstrated the critical roles of KCs in the development of chronic ALD. Chronic alcohol exposure can lead to intestinal hyperpermeability, resulting in the elevation of circulating endotoxin/LPS levels. LPS translocates from gut to liver, leading to the activation of KCs. Activated KCs produce a large amount of ROS, pro-inflammatory cytokines, and chemokines and induce the infiltration of other inflammatory cells, which finally cause liver injury (Figure 1).

### Evidence Demonstrates that KCs Activation by Gut-Derived Endotoxin/LPS Plays Pivotal Roles in the Pathogenesis of Chronic ALD

The number of KCs in portal tract of liver was increased in ALD patients as well as in chronic ethanol-intoxicated animals (13, 14). Parallelly, the levels of many pro-inflammatory cytokines and chemokines secreted by KCs in chronic ethanol-intoxicated animals were significantly increased (15, 16). Furthermore, KCs elimination by gadolinium chloride almost completely prevented chronic ethanol-induced fat accumulation, inflammation, and necrosis scores (17–19). Now, it is clear that chronic ethanol-induced activation of KCs is attributed to gut-sourced LPS, which is a major component of outer membrane of Gram-negative bacteria and passes through the intestinal epithelial barrier in trance amounts under normal condition (20, 21). Chronic ethanol exposure can increase translocation of LPS from gut to liver by enhancing the intestinal permeability and altering the gut microflora. It has been demonstrated that chronic ethanol exposure can induce hemorrhagic lesions and pronounced alteration in the ultrastructure of enterocytes in small intestine of animals and human beings, leading to the increased permeability of intestinal mucosa to macromolecules (22–25). Additionally, chronic ethanol consumption may alter gut microflora, favoring the overgrowth of Gram-negative bacteria and thus increasing the source of LPS (26, 27). Elevation of serum LPS levels was observed in chronic ethanol-feeding rats and also in ALD patients (28). Suppressing LPS-producing bacteria by probiotics significantly reduced the serum LPS level and attenuated liver injury (29). Furthermore, animal studies showed that intestinal sterilization by antibiotics or LPS receptors deficiency significantly suppressed chronic ethanol-induced liver injury (30–32). These studies clearly demonstrate that the activation of KCs by gut-derived LPS plays causal roles in the pathogenesis of chronic ALD.

**Figure 2:**
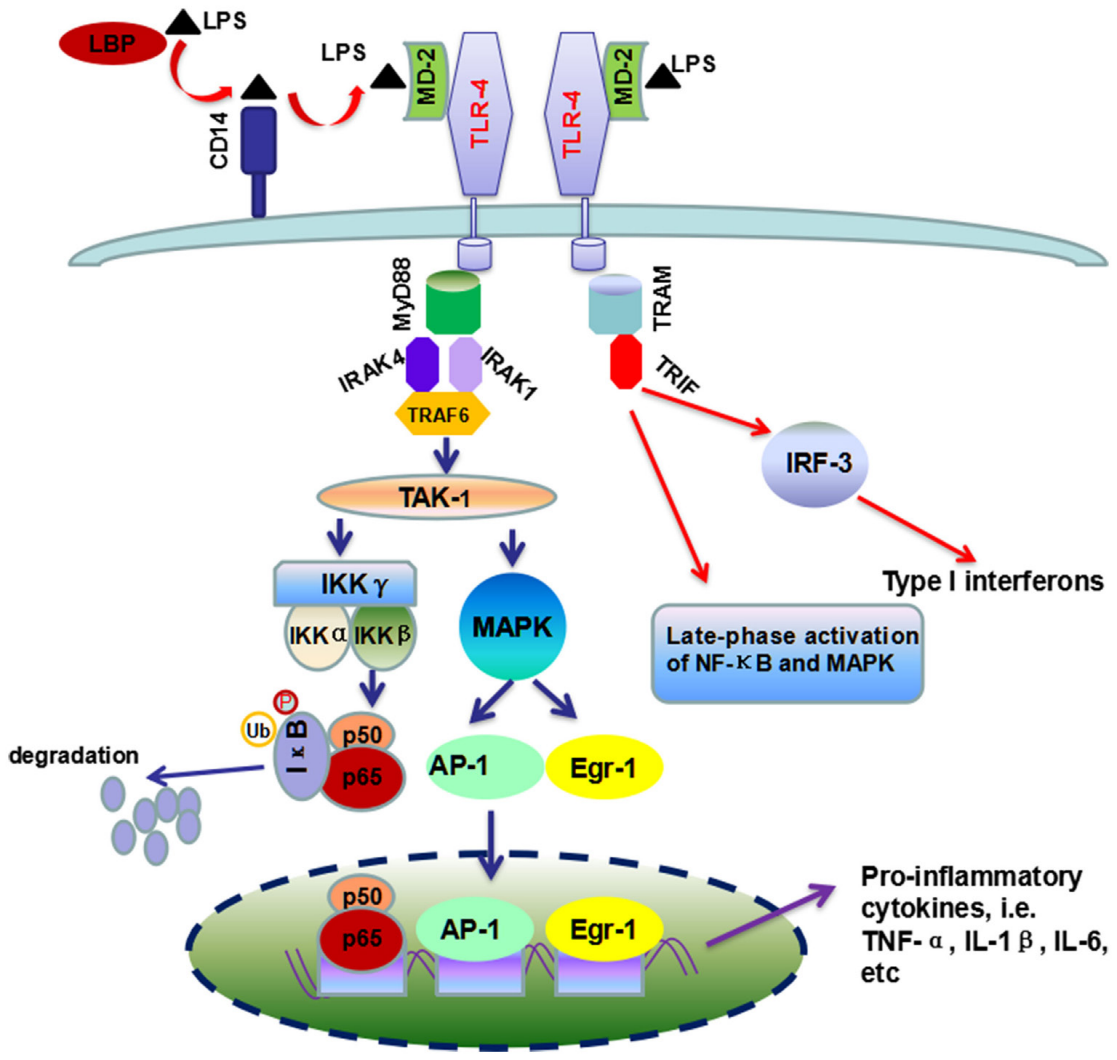
**Overview of TLR-4/LPS signaling pathway activated by LPS in ALD**. Chronic ethanol exposure leads to increased translocation of LPS to liver. In liver, LBP (the shuttle protein) transfers LPS to CD14, which facilitates the binding of LPS to TLR-4/MD-2 complex. TLR4 undergoes dimerization and transduces signal by two different pathways, i.e., MyD88-dependent and TRIF-dependent pathways. The former pathway included the recruitment of IRAK4, IRAK1, and TRAF-6, which ultimately leads to the production of pro-inflammatory cytokines by the activation of NF-κB and MAPK. In the second scenario, TRIF triggers a signaling pathway which controls the production of type I interferon and some other cytokines, as well as the late-phase activation of NF-κB and MAPK.

Signaling studies reveal that LPS can activate the toll-like receptor 4 (TLR-4) in KCs by incorporating in an activation complex involving LPS-binding protein (LBP), cluster of differentiation 14 (CD14) and myeloid differentiation factor 2 (MD-2) (21, 33). LPS is transferred by LBP (a shuttle protein) to CD14 and then binds with TLR-4/MD-2 receptor complex (34, 35). TLR-4 undergoes oligomerization and triggers myeloid differentiation primary response gene 88 (MyD88)- and toll-interleukin-1 receptor domain-containing adaptor inducing interferon-β (TRIF)-dependent production of pro-inflammatory cytokines, and type I interferon (IFN), respectively (33, 36). In the MyD88-dependent scenario, MyD88 recruits downstream adaptors including IL-1 receptor-associated kinase-4 (IRAK-4), IRAK-1, and TNF receptor-associated factor 6 (TRAF-6), leading to the activation of transforming growth factor β-activated kinase 1 (TAK-1) (33, 36, 37). TAK-1 can activate IκB kinase (IKK) and mitogen-activated protein kinase (MAPK) (38). Activated IKK phosphorylates IκB, resulting in the degradation of IκB proteins and the subsequent nuclear translocation of active NF-κB dimmers (39), while MAPK activates the early growth response 1 (Egr-1) and activation protein 1 (AP-1) (33, 40–42). On the other scenario, TRIF initiates a signaling pathway which activates interferon regulatory factor 3 (IRF-3) transcription factor and the late-phase activation of NF-κB and MAPK, leading to the expression of type 1 IFN and IFN-inducible chemokines (Figure 2).

Chronic ethanol exposure led to increased DNA binding activity of NF-κB, and KCs were considered as the major cell type for NF-κB activation in liver (43). Importantly, NF-κB activation preceded the histopathological changes of liver in ALD rats and inactivation of NF-κB by delivery of IκB superrepressor gene with adenovirus significantly reduced chronic ethanol-induced liver injury, indicating the causal roles of NF-κB activation in the pathogenesis of ALD (44, 45). NF-κB may mediate liver injury by enhancing the production of TNF-α, as TNF-α antibodies and TNF receptor 1 (TNFR-1) knockout all significantly reduced chronic ethanol-induced liver injury (46, 47). Interestingly, the activation of NF-κB and subsequent TNF-α production could be suppressed by antioxidants or delivery of superoxide dismutase (SOD), which suggests that ROS may act as signaling molecules for NF-κB activation (48). NADPH oxidase (NOX) and cytochrome P4502E1 (CYP2E1) have been suggested to be major sources of ROS in ALD (49–52). NOX inhibitor (diphenyleneiodonium sulfate, DPI) and loss of p47^phox^ subunit of NOX all significantly blunted the ethanol-induced oxidative stress, NF-κB activity, and TNF-α expression in the liver (53). These results strongly support the hypothesis that ROS from NOX in KCs promotes the NF-κB activation and TNF-α production (54).

### Hepatocytes May Be Sensitized to Chronic Alcohol-Induced Injury

Alcohol may increase the sensitivity of hepatocytes to liver injury by increasing LPS-induced production of TNF-α, modifying cell surface receptors of TNF-α and forming reactive aldehydes adducts.

First, chronic alcohol might increase the sensitivity of KCs to secrete TNF-α and other pro-inflammatory cytokines (55). *In vivo* studies showed that chronic ethanol feeding enhanced LPS-stimulated TNF-α production and liver injury, which could be attenuated by TNF-α synthesis inhibitor (56, 57). *In vitro* studies using isolated KCs suggested that KCs from ethanol-fed rats secreted more TNF-α compared with those isolated from control animals (58–60). Further mechanisms investigation revealed that chronic alcohol exposure increased the half-life of TNF-α mRNA by activating p38MAPK, extracellular signal regulated kinases 1 and 2 (ERK1/2) and Egr-1 in KCs (58, 59, 61). It has been demonstrated that ERK1/2 plays critical roles in mediating the expression of TNF-α, interleukin 1β (IL-1β), etc., as specific inhibitor of ERK1/2 treatment led to the reduction of mRNA levels of these cytokines (40). As DPI could abrogate LPS-induced production of TNF-α as well as the activation of ERK1/2, it is possible that ERK1/2 is an important target of NOX (60). Second, chronic alcohol exposure could markedly elevate the affinity and capacity of binding sites of TNFR on hepatoctyes, and increase the mRNA and/or protein levels of LBP, CD14, and MD-2 in liver tissues, which may sensitize the liver to LPS-induced injury (59, 62–65). Third, chronic alcohol exposure can induce oxidative stress and lipid peroxidation in liver, resulting in overproduction of reactive aldehydes, including malondialdehyde (MDA) and 4-hydroxynonenal (4-HNE), which exhibit reactivity with proteins. It has been demonstrated that MDA can react with acetaldehyde and proteins to form hybrid protein adducts, designated as MAA adducts (66). *In vitro* exposure of KCs to MAA adducts induced pro-inflammatory and profibrogenic response, and MAA could synergistically interact with LPS to increase cytokines and chemokines expression (67).

### CYP2E1 Potentiates Liver Injury Induced by LPS-Activated KCs

Cytochrome P4502E1, a member of the hemo-containing cytochrome P450 superfamily, has been suggested to play important roles in the pathogenesis of ALD. In addition to hepatocytes, CYP2E1 is also expressed in the KCs and in other tissues, including intestine and adipose (68, 69). CYP2E1 is an inducible enzyme and is the major component of the microsomal ethanol-oxidizing system (MEOS) (70–72). The activity of hepatic CYP2E1 in rats exposed to ethanol (5%, w/v)-containing Lieber-DeCarli diet was significantly increased by threefold to fivefold (73). The central roles of CYP2E1 in the pathogenesis of ALD have been highlighted by series of studies in which specific CYP2E1 inhibitors [such as diallyl sulfide and clomethiazole (CMZ)] or genetic knockout of CYP2E1 significantly attenuated chronic ethanol-induced liver injury (3, 49, 74–77). The important role of CYP2E1 in the development of ALD has been attributed to CYP2E1-mediated production of ROS (78–81).

**Figure 3:**
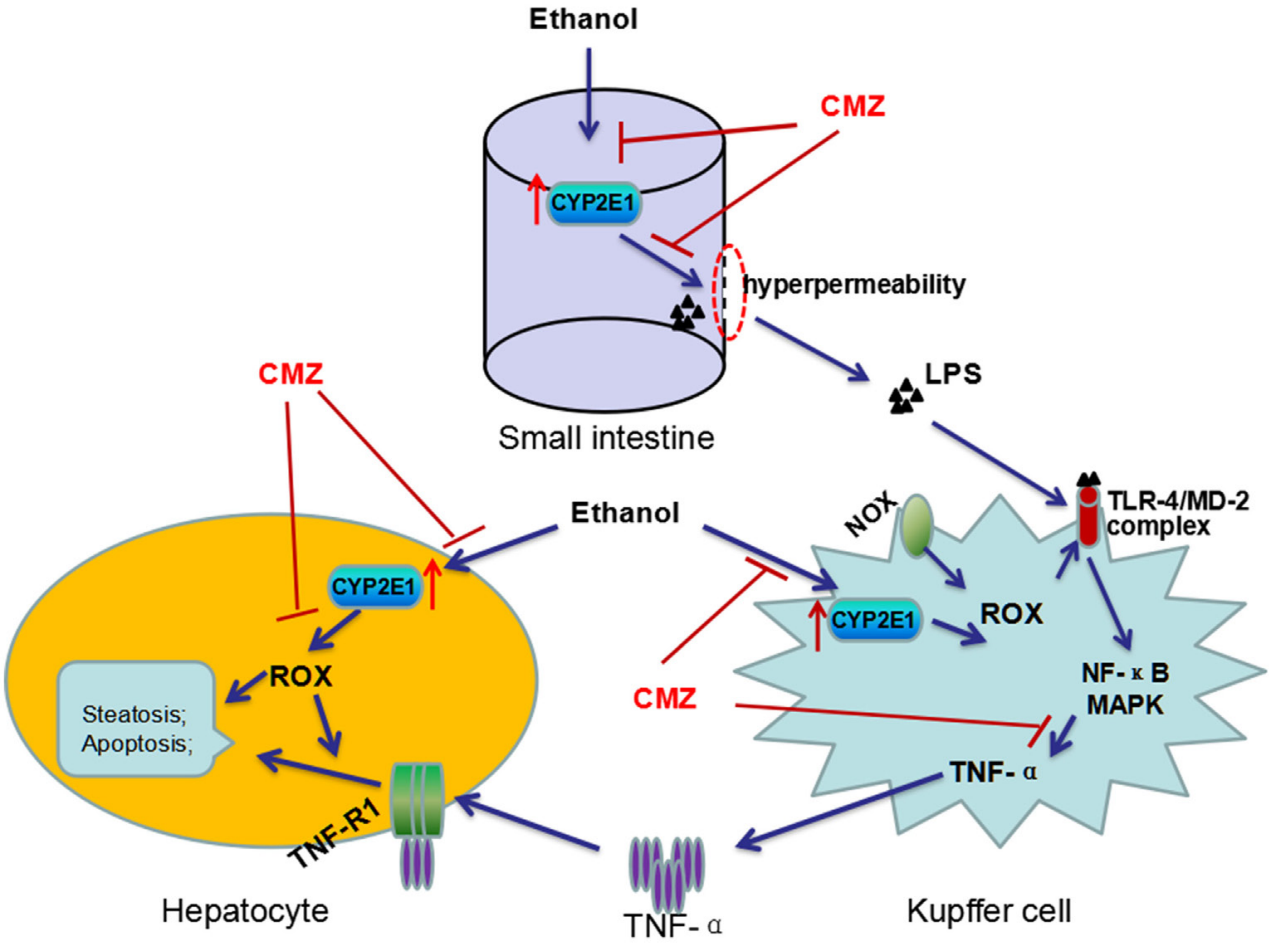
**Interactions between CYP2E1 and LPS/TNF-α**. CYP2E1 has been demonstrated to be expressed in hepatocytes, KCs, and also in small intestines. Intestinal CYP2E1 activation promotes ethanol-induced gut hyperpermeability, while CYP2E1 in KCs potentiates LPS-induced production of TNF-α. Furthermore, CYP2E1 can sensitize the hepatocytes to LPS/TNF-α toxicity from proliferation to apoptotic and necrotic cell death. All these adverse effects of CYP2E1 can be suppressed by CMZ, a specific inhibitor of CYP2E1.

Interestingly, LPS/TNF-α and CYP2E1, the two contributors to ALD, are not exclusive of each other, as a number of studies have revealed interactions between CYP2E1 and LPS/TNF-α (20, 73). First, activation of CYP2E1 in hepatocytes sensitizes the hepatocytes to LPS/TNF-α toxicity (82). CYP2E1 overexpression converted the hepatocyte TNF-α response from proliferation to apoptotic and necrotic cell death *via* activating JNK, which can be suppressed by antioxidants (83). Combination exposure to ethanol and TNF-α resulted in more serious cytotoxicity to CYP2E1-expressing HepG2 cells than the wild-type HepG2 cells (84). *In vivo* studies showed that chronic alcohol feeding enhanced LPS-stimulated TNF-α production and liver injury in wild-type mice, while CYP2E1^−/−^ mice appeared to be resistant to LPS-induced hepatotoxicity (49, 56, 57). In addition, chronic alcohol exposure can lead to CYP2E1 activation in small intestine as well as in KCs (85, 86). Studies regarding the intestinal CYP2E1 showed that intestinal CYP2E1 might promote ethanol-induced intestinal hyperpermeability *via* a mechanism involving induction of oxidative stress and upregulation of circadian clock proteins CLOCK and PER2 (52, 85, 87). In KCs, CYP2E1 could induce more TNF-α secretion after stimulation by LPS, which was significantly blunted by CYP2E1 inhibitor, CMZ (86). Take together, CYP2E1 may play multiple roles in the pathogenesis of ALD, such as increasing ROS production, enhancing intestinal permeability, and promoting the TLR-4/LPS signaling to produce more TNF-α (Figure 3).

### The Roles of Inflammasome and IL-1β in the Pathogenesis of ALD

Inflammasomes are intracellular multiprotein complexes in both parenchymal and non-parenchymal cells activated upon cellular infection or stress that trigger the activation of caspase-1 and release of pro-inflammatory cytokines (88–90). Inflammasome activates procaspase-1 generating IL-1β, which causes inflammation in an autocrine fashion *via* IL-1 receptor (11). IL-1β, produced in inactive pro-IL-1β, is an important pro-inflammatory mediator in ALD. Inflammatory stimuli can induce the expression of inactive pro-IL-1β, and also can drive the formation of inflammasome leading to the cleavage of pro-IL-1β into the bioactive IL-1β (88, 91). It has been found that the serum levels of IL-1β in ALD patients and chronic ethanol-treated rats were significantly increased compared with controls (92, 93). In a mouse model of alcoholic hepatitis (AH), Petrasek et al. found that inflammasome was activated in KCs of ALD mice, which promoted caspase-1-mediated activation of IL-1β (91). Importantly, *in vivo* intervention with a recombinant IL-1 receptor antagonist (IL-1Ra) blocked IL-1β signaling and markedly attenuated ethanol-induced inflammation, steatosis, and liver damage (91). Results of these studies suggest that inflammasome activation is a component of the liver pathophysiology in ALD (89, 94, 95).

## Roles of KCs in Acute Ethanol/Binge Drinking-Induced Liver Injury

### Biphasic Effect of Acute Ethanol/Binge Drinking on the Activation of KCs

A binge drinking is defined as consumption of five and four drinks for men and women, respectively, in 2 h to produce a blood ethanol level over 80 mg/dl (96). Binge drinking is on the rise at an alarming rate worldwide (97–99). Although acute and chronic ALD may share similar mechanisms, the deleterious effects of alcohol may be affected by the doses and duration of ethanol (100). In contrast to the increased inflammation induced by chronic ethanol consumption, acute/binge drinking and regular moderate drinking has been reported to exert anti-inflammatory effects (96, 101). Numerous studies have demonstrated that acute/binge drinking (2–7 g/kg body weight, by introperitoneal injection or by gavage) could significantly suppress bacteria, LPS, and TLR ligand-induced increase of TNF-α levels in serum, bronchoalveolar lavage (BAL), and peritoneal lavage fluid (56, 102–111). For example, Nelson et al. found that acute ethanol (5.5 g/kg body weight, by introperitoneal injection), but not chronic ethanol consumption, markedly inhibited LPS-induced increase of TNF-α level in serum and BAL, and also suppressed systemic and intrapulmonary polymorphonuclear leukocyte aggregation (103). Honchel et al. found that acute ethanol (2 g/kg body weight, by gavage) suppressed LPS-induced TNF-α production, while chronic ethanol led to further elevation of serum TNF-α and significant liver injury (56). The anti-inflammatory effect of binge drinking was also examined in *in vitro* studies. Acute ethanol intoxication significantly suppressed LPS-induced elevation of inducible nitric oxide synthase (iNOS) mRNA in KCs and reduced LPS-induced serum TNF-α activity and ROS release (104, 112). Results of these studies seem to suggest that acute/binge drinking induce inactivation and tolerance of KCs to LPS, which is paradoxical to the effects of chronic ethanol exposure. However, time-effects studies revealed that acute/binge drinking might cause transient tolerance of KCs to LPS, followed by the enhanced sensitivity to LPS. For example, the study by Enomoto et al. found that KCs isolated from rats exposed to acute ethanol (4 g/kg body weight) 2 h ago exhibited decreased release of TNF-α, while KCs isolated from rats exposed to acute ethanol 24 h ago displayed enhanced secretion of TNF-α (113). Similarly, the study by Yamashina et al. revealed that ethanol administration at 1 h before LPS exposure alleviated LPS-induced liver injury, while ethanol administration at 21 h before LPS aggregated liver injury. This biphasic effect of ethanol was correlated with the expression of IRAK, NF-κB, and the TNF-α levels (114).

### Evidence Suggests that KCs Play Crucial Roles in Acute Ethanol/Binge Drinking-Induced Liver Injury

Although limited, several studies have suggested that KCs may play crucial roles in acute ethanol/binge drinking-induced liver injury (52, 100, 115, 116). Binge drinking (6 g/kg body weight) could also lead to transient increase of the serum endotoxin/LPS levels (115, 116). Interestingly, endotoxin-neutralizing protein abrogated binge drinking-induced increase of hepatic TNF-α level, oxidative stress, and liver injury (115). Using a more stringent binge drinking mice model (mice exposed to three doses of ethanol, 6 g/kg body weight each, at 12 h intervals), wild-type mice in ethanol group developed hepatic steatosis and significant increase of serum endotoxin/LPS level as well as hepatic enterobacteria level (52). As binge drinking-induced TNF-α production and liver injury could be effectively attenuated by antioxidant (*N*-acetylcysteine) and specific inhibitor of CYP2E1 (chlormethiazole), it could be concluded that oxidative stress might mediate endotoxin/LPS-induced TNF-α production in acute ethanol-induced liver injury (52, 115). Furthermore, probiotics, which could maintain the intestinal integrity, significantly suppressed acute ethanol-induced liver injury (117). Results of these studies suggest that the “gut–liver axis” plays crucial roles in acute/binge drinking-induced liver injury.

To test the roles of gut microbiota in the pathogenesis of acute ALD, the sensitivity to binge drinking (3 g/kg body weight)-induced liver injury was compared between germ-free and conventional C57BL/6 mice (118). Unexpectedly, germ-free mice were found to be more susceptible to binge drinking-induced hepatic fat accumulation, elevation of serum aminotransferase activity, and hepatic inflammation compared with the conventional mice. The germ-free animals exhibited higher basal levels of hepatic fat content and CYP2E1 protein level compared with the conventional mice, which might explain the increased sensitivity to binge drinking-induced liver injury (118). As complete loss of intestinal microbiota seems to affect the metabolic homeostasis of mice liver, it may be not appropriate to use these germ-free animals to test the “gut–liver axis” theory (119).

## The Polarization of KCs and the Pathogenesis of ALD

### The Polarization of KCs in ALD

**Figure 4:**
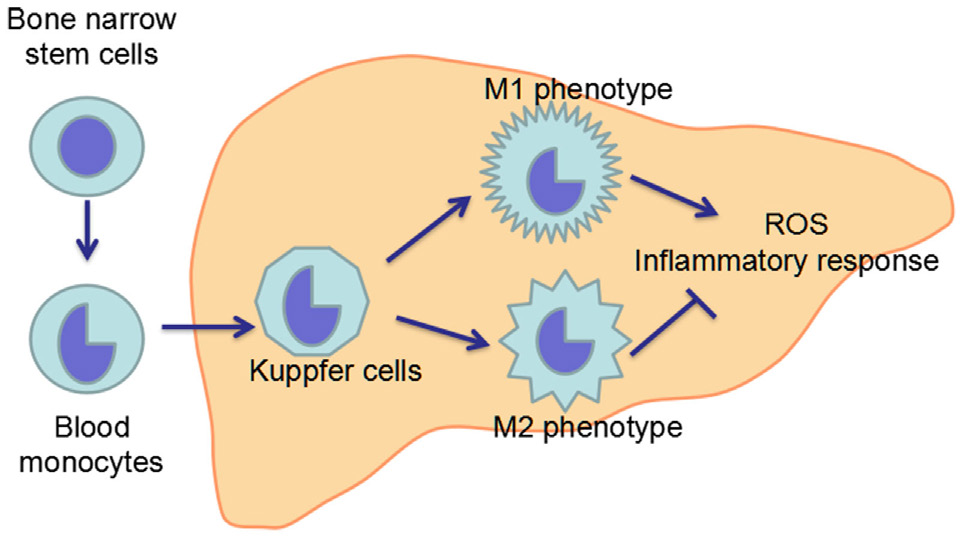
**Maturation and polarization of KCs**. KCs, the resident macrophage in the liver, originate from the precursor cells in the bone marrow, which give rise to blood monocytes. Blood monocytes migrate into liver and develop in to liver macrophage, namely KCs. In the liver, KCs can polarize in two ways: classical activation/M1 polarization and alternative activation/M2 polarization, which exhibit pro-inflammatory and anti-inflammatory effects, respectively. The imbalance between M1 and M2 polarization of KCs contributes to the pathogenesis of ALD.

Macrophages including KCs are exceptionally plastic cells which can polarize to specific activation state and express different functions in response to microenvironmental signals. Two well-established polarized phenotypes are often referred to as classically activated macrophage (M1 polarization) and alternatively activated macrophage (M2 polarization) (120, 121). The nomenclature of M1/M2 polarization is derived from the cytokines that are associated with these macrophage phenotypes as these cytokines, namely interferon γ (IFN-γ) or interleukin-4 (IL-4), are linked with T helper 1 (Th1)- and Th2-type immune responses, respectively (120, 122). M1-polarized macrophages are characterized by increased expression of pro-inflammatory cytokines, including TNF-α and iNOS, while M2-polarized macrophages exhibit low expression of pro-inflammatory cytokines, but increased expression of anti-inflammatory mediators, such as IL-10 (4, 12). Inflammatory stimuli such as microbes, damaged tissues, and activated lymphocytes, can induce macrophages to acquire the pro-inflammatory M1 polarization (123). The inflammation driven by the M1 macrophages is counterbalanced by the anti-inflammatory M2-polarized macrophages which can promote the inflammation resolution and tissue repair (124) (Figure 4).

Both chronic ethanol exposure and acute/binge drinking can lead to increased hepatic translocation of LPS, which is a strong inducer of M1 activation of KCs (125, 126). Chronic ethanol feeding has been demonstrated to result in increase of both M1 markers genes [*iNOS, tumor necrosis factor* α (*TNFA*) and *monocyte chemoattractant protein-1* (*MCP1*)] and M2 markers genes [*Arginase 1* (*Arg1*), *mannose receptor C type 2* (*Mrc2*)] in mice liver, but the induction of M1 marker genes was more profound than the M2 marker genes (127, 128). The imbalance between M1 and M2 polarization leads to the overproduction of pro-inflammatory cytokines, which finally induce hepatocytes injury. Although there is no direct study about the polarization of KCs in acute/binge drinking animal models, it could be speculated that KCs may also undergo M1 polarization after acute ethanol exposure.

### KCs Polarization toward the Alternative State (M2 Phenotype) May Be an Attractive Strategy for ALD Treatment

As macrophages of M1 and M2 phenotypes are not stably differentiated subsets, pharmacological interventions targeting M2 polarization during early stages of ALD may represent an attractive strategy to limit ethanol-induced inflammation and hepatocytes injury (5). Indeed, regulation of M1/M2 balance in KCs by agonist of cannabinoid CB2 receptor (a G-protein-coupled receptor predominantly expressed by cells of the immune system) significantly suppressed chronic ethanol-induced hepatic steatosis (127). In a recent study, Wan et al. found that KCs in ALD-resistant BALB/c mice displayed preponderant M2 polarization compared to KCs in ALD-sensitive C57BL6/J mice, and ongoing alcohol drinkers with minimal hepatic injury displayed higher hepatic expression of M2 genes as compared to patients with severe liver lesions (128). Mechanisms studies showed that M2-polarized KCs promoted death of selective M1-polarized KCs by producing IL-10 and triggered hepatocytes senescence (128, 129). Furthermore, many studies have demonstrated that ω-3 polyunsaturated fatty acids (PUFAs) such as docosahexaenoic acid (DHA) and DHA-rich fish oil could attenuate ALD (130–132). As unsaturated fatty acids and saturated fatty acids could induce macrophages M2 activation and M1 activation, respectively (133–135), it would be possible that these PUFAs may confer protection against ALD by modulating the polarization of KCs.

## Potential Therapeutic Targets for ALD Treatment Related with Gut Endotoxin/LPS–Liver KCs Axis

The critical roles of KCs and related inflammatory cascade in the pathogenesis of ALD make it a promising target in pharmaceutical drug developments for ALD treatment. Several potentially therapeutic drugs, including antibiotics, probiotics, anti-TNF-α, and anti-IL-1β agents, have been evaluated or are under evaluation for the efficacy in ALD treatment in animal studies and/or clinical trials. Specially, several anti-TNF-α agents, including pentoxifylline, infliximab, and etanercept, have been tested for the treatment of severe AH. Unfortunately, although animal studies usually showed promising effects, clinical trials have brought conflicting results.

### Antibiotics

Theoretically, antibiotics can block the development of ALD, as gut-derived endotoxin/LPS initiates the TLR-4 signaling pathway, leading to the production of pro-inflammatory cytokines. Indeed, animal studies showed that antibiotics (polymyxin B and neomycin) completely prevented the elevation of aspartate aminotransferase activity and significantly reduced the average hepatic pathological scores in ethanol-exposed rats (30). Rifaximin, a non-absorbed antibiotic with few side effects and little evidence for resistance, has been evaluated for the treatment of alcoholic cirrhosis in clinical trials (136). One study found that rifaximin (1200 mg/day) could improve thrombocytopenia in patients with alcoholic cirrhosis (137). Portal hypertension is crucial in the transition from the compensated phase to the decompensated phase of cirrhosis, as it is associated with the most severe complication of cirrhosis, including hepatorenal syndrome (138, 139). Results of two studies showed that intestinal decontamination with rifaximin (1200 mg/day) for 4 weeks led to significant decrease of hepatic venous pressure gradient (HVPG), cardiac output, and plasma rennin activity, accompanied with significant increase of glomerular filtration rate and natriuresis (140, 141). A study suggested that long-term rifaximin administration (1200 mg/day, followed up for 5 years) might be associated with reduced risk of developing complications of portal hypertension and improved survival in patients with alcohol-related decompensated cirrhosis (142). Results of these studies suggest that rifaximin may be beneficial for the reduction in portal pressure and improvement of renal function in decompensated alcoholic cirrhotic patients. These results are quite promising, and are needed to be further evaluated by well-designed randomized-controlled trials with larger sample size.

### Probiotics

Probiotics are defined as “live microorganism which can confer health benefits on the host when administered in adequate amounts” (143). Probiotics are non-pathogenic beneficial flora that act to regulate and maintain a stable intestinal environment and promote micro-ecological balance (144). The beneficial effects of probiotics against ALD have been gaining increased interest in recent years. Many probiotics such as *Lactobacillus rhamnosus* and probiotic mixtures VSL#3 have been demonstrated to protect against both chronic and acute ALD in animal studies (117, 145–152). The mechanisms included the improvement of intestinal integrity, reduction of TLR-4 expression, and activation of AMP-activated protein kinase (AMPK) (117, 149, 151, 152). A pilot prospective, randomized, clinical trial showed that probiotics supplementation could restore bowel flora and improve liver enzymes in ALD patients (153). A randomized-controlled multicenter study showed that 7 days of oral supplementation with probiotics restored the bowel flora and improved the LPS in patients with AH (29). Results of these studies provide a scientific rationale to further test probiotics for treatment and/or prevention of ALD in humans.

### Anti-TNF-α Agents

Several anti-TNF-α agents have gained great interest due to the important roles of TNF-α in the pathogenesis of AH. Three anti-TNF-α agents have been investigated for AH treatment, i.e., pentoxifylline, infliximab, and etanercept. However, the clinical use of these agents is yet not recommended due to the poor clinical outcomes observed in the largest clinical trials.

Pentoxifylline is a non-selective phosphodiesterase inhibitor, which can inhibit endotoxin-induced production of TNF-α (154). A pilot study showed that pentoxifylline (400 mg × 3 times/day for 28 days) significantly reduced the mortality of severe AH (46 vs. 25%, *p* = 0.037); while another study showed pentoxifylline treatment significantly improved the renal and hepatic functions accompanied with a trend of decreasing mortality as compared with the placebo group (155, 156). However, the studies comparing the efficacy between pentoxifylline and corticosteroid (the first-line treatment for severe AH) have produced conflicting results. One study found that pentoxifylline was superior to corticosteroid in reducing the mortality and hepatorenal syndrome (157), while another showed that the efficacy of the pentoxifylline was not statistically equivalent to that of corticosteroid (158). In addition, the combination of pentoxifylline and corticosteroid did not significantly improve the 28-day and 6-month survival of severe AH patients when compared with treatment with corticosteroid alone (159). A recently published multicenter, double-blind, randomized trials with a 2-by-2 factorial design showed that pentoxifylline did not improve survival of AH patients (160). Due to these conflicting results, several meta-analyses have been performed, and the results showed that pentoxifylline could not improve the short-term survival of AH patients compared with placebo; however, combination of pentoxifylline and corticosteroid could reduce the incidences of hepatorenal syndrome and the infection risk compared with corticosteroid therapy alone (161–163).

Infliximab is a monoclonal human/mouse anti-TNF-α antibody which can bind to TNF-α with high affinity when administered by intravenous infusion. In contrast to the efficacy observed in the treatment of rheumatoid arthritis and Crohn’s disease, the efficacy of infliximab in the treatment of AH is controversial. Animals study showed that infliximab at 1 mg/kg body weight exhibited immunomodulatory and anti-inflammatory effects, whereas infliximab at 10 mg/kg body weight significantly decreased lipid accumulation in experimental AH model (164). An earlier pilot clinical trial found that serum bilirubin levels, Maddrey scores, neutrophil counts, and C-reactive protein levels fell significantly within the first month in patients receiving single dose of infliximab (5 mg/kg body weight) (165). A randomized-controlled clinical trial in 20 biopsy-proven AH patients showed that combination of single dose of infliximab (5 mg/kg body weight) and prednisone (40 mg/day for 28 days) improved the Maddrey scores and laboratory parameters, while prednisone treatment alone had no significant alteration on these parameters (166). However, another multicenter double-blind randomized-controlled trial of infliximab associated with prednisolone in 36 AH patients found that the survival rate and frequency of severe infections within 2 months was higher in infliximab and prednisolone group than in the placebo and prednisolone group (167). The study was stopped by the follow-up committee and the sponsor at the end of 2 months, as more deaths occurred in infliximab co-treatment groups. Another study showed that a single dose of infliximab (5 mg/kg, i.v.) was associated with significant improvement in parameters of severity, but failed to suppress the infection (168).

Etanercept is a genetically engineered, soluble, systemic TNF-α blocker that competitively binds to and neutralizes both soluble and transmembrane forms of TNF-α (169). A pilot study examining the effects of etanercept in 13 patients with moderate to severe AH showed that the survival rate of 1 month was 92% (12/13), although 23% of patients (3/13) develop serious adverse effects including infection, hepatorenal decompensation, and gastrointestinal bleeding (170). However, a multicenter, randomized, double-blind, placebo-controlled study in 48 patients with moderate to severe AH (defined as model for end-stage liver disease score ≥15) found that etanercept (given six times over 3 weeks) did not significantly affect the 1-month survival rates compared with placebo treatment, while the 6-month mortality rate was significantly higher in the etanercept group compared with the placebo group (57.7 vs. 22.7%, *p* = 0.017) (171).

### IL-1β Signaling Inhibitors

The key role of inflammasome activation in ALD progression has been confirmed using experimental mouse models. Activation of inflammasome leads to the production of pro-inflammatory cytokine IL-1β, which has been demonstrated to play critical roles in alcoholic steatohepatitis (95). IL-1β signals through IL-1R, leading to inflammatory cascade. IL-1Ra is a naturally occurring cytokine that binds to IL-1R to regulate the actions of IL-1β and control inflammation (172). Animal studies have shown that recombinant IL-1Ra could significantly attenuate ethanol-induced liver inflammation and injury (91, 172). As anti-TNF-α agents did not bring convincing results in some clinical trials, IL-1Ra has gained great interest, and a clinical trial is currently underway to test the efficacy of IL-1Ra (Anakinra) in the treatment of AH (173).

### Other Drugs Potentially Used for ALD Treatment

In addition to the above referred drugs for ALD treatment, the gut–liver axis also provides many other potentially therapeutic targets, including intestinal permeability maintaining agents, LPS antibodies, TLR-4 antagonist, and caspase inhibitors. The efficiency of these drugs has been examining in several ongoing clinic trials (11).

## Conclusion and Remarks

The causal roles of KCs in the pathogenesis of ALD have been highlighted, and the underlying molecular mechanisms have been revealed. KCs are activated in both chronic and acute ALD, which is driven by gut-sourced endotoxin/LPS. LPS mediates activation of TLR-4 signaling pathway in classically activated KCs (M1 phenotypes), leading to the overproduction of ROS, pro-inflammatory cytokines, and chemokines. The increased release of pro-inflammatory cytokines and the infiltration of other inflammatory cells such as the neutrophils finally cause liver injury.

The “gut–liver axis” suggests that suppression of KCs activation (such as suppressing the production of LPS, reducing the combination of LPS with TLRs, etc.) and elimination of cytotoxic products secreted by KCs (such as using TNF-α antibodies and IL-1Ra) may be beneficial to the treatment of ALD. Several drugs (such as rifaximin, pentoxifylline, and infliximab) have been evaluated or are under evaluation for ALD treatment in randomized clinical trials. Although animal studies usually showed beneficial effects, conflicting results were obtained from studies about the anti-TNF-α agents in AH treatment, which might be related with differences in the study design, the enrollment criteria and the baseline characteristics of AH patients, and the ethnicity of study population. For example, although the same threshold of AH (Maddrey scores ≥ 32) was used in all the trials, the baseline characteristics of patients such as the age and rate of encephalopathy in these trials were not consistent, which might influence the accuracy of the results as these characteristics could influence mortality (160, 174, 175). Importantly, the study by Petrasek et al. suggests the therapeutic potential of IL-1β inhibitors for the treatment of ALD. It is urgent to investigate whether IL-1β inhibitors are beneficial for AH patients, as severe AH is associated with high mortality and lack of effective treatments (172, 176). Furthermore, the study by Wan et al. provided solid evidence for the protective roles of M2 KCs against early stage of ALD (128). Thus, screening pharmacological regulators for KCs toward M2 polarization may provide additional therapeutic agents. The combination of these potentially therapeutic drugs with other agents (such as hepatoprotective agents, including zinc, melatonin, silymarin, etc.) may bring encouraging results.

Although the roles of KCs in the pathogenesis of ALD have been clearly demonstrated, there are still some questions that need to be addressed. First, the autophagic response of KCs in ALD remains unclear. Autophagy, an intracellular self-digestion process, can regulate lipid metabolism in hepatocytes and also have anti-inflammatory effects (177, 178). Activation of autophagy could reduce alcoholic and metabolic steatosis by enhancing the decomposition of lipid and suppress LPS-induced inflammation and liver injury (178–181). Although ethanol exposure induces autophagy in the mice/rat livers and in cultured hepatocytes, no studies investigated the autophagic response of KCs in ALD (182, 183). It has been demonstrated that LPS can induce autophagy in human and murine macrophages (184, 185). Therefore, it appears to be reasonable that ethanol may also lead to the activation of autophagy in KCs as ethanol could increase LPS translocation from gut to liver. The autophagic response of KCs and its roles in ALD are needed to be investigated. Second, the relationships between autophagy and polarization in KCs are needed to be elucidated. One previous study showed that KCs from mice with Atg 5 deficiency in macrophages exhibited increased M1 polarization and decreased M2 polarization, suggesting that autophagy has a critical regulatory function in macrophage polarization (178). It should also be necessary to explore whether KCs polarization regulator can influence autophagy activity. Third, the highly heterogeneous KCs can be classified into different subpopulations. For example, two F4/80+ KCs subsets have been revealed, a CD68+ subset with phagocytic activity and a CD11b+ subset with cytokine-producing capacity (6, 186). These different KC subsets may play different roles in the pathogenesis of ALD, which need to be further studied.

In addition to ALD, KCs activation may be also involved in the pathogenesis of alcohol dependence, which is characterized by an individual’s continued drinking despite negative consequences related to alcohol use (187–190). It was found that intestinal permeability and blood LPS were largely increased in alcohol-dependent subjects and the pro-inflammatory cytokines level was positively correlated with craving (191). Evidences from postmortem alcoholic brain showed that chronic ethanol could increase degradation of tight junctions and extracellular matrix in human brain (192). Additionally, animal study demonstrated that TNF-α produced by KCs could transfer to the brain through TNF-R to induce the synthesis of additional TNF-α, creating a persistent and self-propelling neuroinflammation in the brain (193). Results of these studies suggest that the cytokines produced by KCs may cross the blood–brain barrier (BBB) *via* diffusion or active transport, or by BBB alterations (189, 190). Further studies about the roles of KCs in the pathogenesis of alcohol dependence may reveal new therapeutic targets for pathologic drinking behaviors.

## Author Contributions

TZ and K-QX designed the study. TZ, C-LZ, MX, RY, and K-QX contributed to the literature search, interpretation, writing, and proofreading of the manuscript. TZ generated the figures.

## Conflict of Interest Statement

The authors declare that the research was conducted in the absence of any commercial or financial relationships that could be construed as a potential conflict of interest.

## Abbreviations

4-HNE, 4-hydroxynonenal; AH, alcoholic hepatitis; ALD, alcoholic liver disease; AMPK, AMP-activated protein kinase; AP-1, activation protein 1; BAL, bronchoalveolar lavage; CD14, cluster of differentiation 14; CYP2E1, cytochrome P4502E1; CMZ, clomethiazole; DHA, docosahexaenoic acid; DPI, diphenyleneiodonium sulfate; Egr-1, early growth response 1; ERK1/2, extracellular signal regulated kinases 1 and 2; IFN, interferon; IKK, IκB kinase; IL-1β, interleukin 1β; IL-10, interleukin 10; IL-1Ra, IL-1 receptor antagonist; iNOS, inducible nitric oxide synthase; IRAK-4, IL-1 receptor-associated kinase-4; IRF-3, interferon regulatory factor 3; KCs, Kupffer cells; LPS, lipopolysaccharide; LBP, LPS-binding protein; MAPK, mitogen-activated protein kinase; MDA, malondialdehyde; MD-2, myeloid differentiation factor 2; MEOS, microsomal ethanol-oxidizing system; MyD88, myeloid differentiation primary response gene 88; NF-κB, nuclear factor kappa B; NOX, NADPH oxidase; PUFAs, polyunsaturated fatty acids; ROS, reactive oxygen species; SOD, superoxide dismutase; TAK-1, transforming growth factor β-activated kinase 1; TNF-α, tumor necrosis factor α; TNFR, tumor necrosis factor receptor 1; TLR-4, toll-like receptor 4; TRIF, toll-interleukin-1 receptor domain-containing adaptor inducing interferon-β; TRAF-6, TNF receptor-associated factor 6.
